# Complete Genome Sequence of the Dairy Isolate *Lactobacillus acidipiscis* ACA-DC 1533

**DOI:** 10.1128/genomeA.01533-16

**Published:** 2017-01-26

**Authors:** Maria Kazou, Voula Alexandraki, Bruno Pot, Effie Tsakalidou, Konstantinos Papadimitriou

**Affiliations:** a Laboratory of Dairy Research, Department of Food Science and Human Nutrition, Agricultural University of Athens, Athens, Greece; b Research Group of Industrial Microbiology and Food Biotechnology (IMDO), Vrije Universiteit Brussel, Brussels, Belgium

## Abstract

*Lactobacillus acidipiscis* is a Gram-positive lactic acid bacterium belonging to the *Lactobacillus salivarius* clade. Here, we present the first complete genome sequence of *L. acidipiscis* isolated from traditional Greek Kopanisti cheese. Strain ACA-DC 1533 may play a key role in the strong organoleptic characteristics of Kopanisti cheese.

## GENOME ANNOUNCEMENT

*Lactobacillus acidipiscis* belongs to the *Lactobacillus salivarius* clade according to the phylogenetic analysis based on the 16S rRNA gene sequence of the *Lactobacillus* genus (1). The clade mainly includes commensals isolated from the intestine/feces of vertebrates and, to a lesser degree, strains isolated from fermented foods (2, 3). *L. acidipiscis* was originally found in fermented fish (4), while strain ACA-DC 1533 was isolated from a 3-month-old traditional Greek Kopanisti cheese prepared from raw cow milk (5). Kopanisti cheese has an intense salty and distinct piquant flavor (6) and strain ACA-DC 1533 may play a key role in the strong organoleptic characteristics of the cheese due to the production of alcohols and carbonyl compounds as major volatile compounds, presumably during secondary amino acid catabolism (5, 7).

Whole-genome sequencing was performed using the Illumina HiSeq 2000 and PacBio RS II platforms at the Beijing Genomics Institute (BGI Co., Ltd., Hong Kong). The libraries used were three Illumina paired-end (500 bp, 2,000 bp, and 6,000 bp inserted size) and one PacBio mate pair 5k/6k. To estimate the genome size, k-mer analysis was performed. Afterward, SOAPdenovo v2.04 software was employed to assemble the reads after filtering, while SOAPsnp, SOAPindel, and GATK were applied for error correction. Furthermore, whole-genome optical mapping generated at Microbion SRL (Verona, Italy) was used to validate the hybrid assembly (8). The alignment of the assembly against the optical map was created with the Argus Optical Mapping System (OpGen Technologies, Inc., Madison, WI). The analysis of the ACA-DC 1533 genome resulted in one circular chromosome of 2,607,423 bp with G+C content of 39.8%. Three plasmid sequences were also detected. Two of them were incomplete and are still under sequencing (data not shown) while the third was plasmid pLAC1 described previously (9).

The chromosomal sequence of ACA-DC 1533 was annotated with RAST v2.0 (10) and prediction of genes was carried out using Prodigal (11), MetaGeneAnnotator (12), and FGENESB (13). GenePRIMP was used for the identification of gene anomalies and putative pseudogenes (14). Manual curation of genes was performed using Artemis (15) and BLAST suite (16). The WebMGA server was used for the identification of genes with Pfam domains (17), whereas signal peptides and transmembrane helices were predicted with the Phobius web server (18). A total of 2,394 genes were annotated in the chromosome of ACA-DC 1533 including 2,262 protein-coding genes, 132 potential pseudogenes, 18 rRNA genes, and 63 tRNA genes. Considering the percentage of potential pseudogenes (approximately 5.5%), it seems that the bacterium has undergone genome decay to an extent, perhaps indicating adaptation to a nutrient-rich environment like that of cheese. The chromosome of ACA-DC 1533 also contains 1,510 protein-coding genes with Pfam domains, 153 with signal peptides, and 427 with transmembrane helices. Further investigation of ACA-DC 1533 may be required to test the applicability of the strain as a starter or adjunct culture according to its technological and probiotic potential.

### Accession number(s).

The chromosomal sequence of *L. acidipiscis* ACA-DC 1533 was deposited at the European Nucleotide Archive under the accession number LT630287.
